# Study to correlate the parental judgement of neonatal jaundice using clinical and biochemical profile

**DOI:** 10.6026/973206300214663

**Published:** 2025-12-15

**Authors:** Aparna Patidar, Nada Ali Imran, Aapurti Awasthi, Rajesh Tikkas

**Affiliations:** 1 Department of Pediatrics, NNF Fellowship Neonatology, Consultant Pediatrician at Ace Clinic, Rohini, New Delhi, India; 2 Department of Pediatrics, Consultant Pediatrician at Kushinagar, Uttar Pradesh, India; 3 Department of Pediatrics, Mahavir Institute of Medical Sciences, Bhopal, Madhya Pradesh, India; 4 Department of Pediatrics, Gandhi Medical College, Bhopal, Madhya Pradesh, India

**Keywords:** Neonatal jaundice, Kramer's grade, clinician, parents

## Abstract

Neonatal jaundice is very common, affecting about 60-80% of newborns, yet delay in recognition still leads to preventable complications. In our study of 500 babies, most were males from rural background and middle or upper-lower socioeconomic class, with 81% reaching hospital within 72 hours. Educated mothers, those from urban homes or with previous history of neonatal jaundice, noticed yellow discolouration earlier and graded jaundice almost similar to clinicians, leading to faster presentation. Thus, we show the accuracy of maternal recognition and grading of neonatal jaundice correlates with clinical assessment and timing of presentation.

## Background:

Neonatal jaundice is extremely common in the early newborn period and reflects bilirubin accumulation during transition to extrauterine life in most healthy babies [1]. It affects about 60-80% of newborns and is a major reason for admission or re-admission in the first postnatal week [2, 3]. Nearly 3% of otherwise healthy term neonates develop total serum bilirubin levels >15 mg/dL [4]. Many times the mother is the first person to notice yellow discolouration, especially when the baby is already discharged home [4, 5]. This early recognition becomes more critical in low- and middle-income countries where out-of-hospital deliveries are still common and multiple risk factors for bilirubin-induced neurological damage remain prevalent [6]. Delayed recognition of neonatal jaundice and late presentation to hospital still remain major contributors to bilirubin encephalopathy, poor neurodevelopmental outcome and sometimes death [2]. Strengthening maternal education and empowerment is one of the most practical ways to improve early identification and intervention, especially in community and home-based settings [5, 7]. Most neonatal jaundice is physiological, but a small proportion progresses to pathological hyperbilirubinemia with risk of permanent neurological injury or even death. Early diagnosis and correct management are therefore not optional but essential. Parents, especially mothers are usually the first to judge jaundice at home and their decision to seek medical care often decides the final outcome. Studying how parental judgement correlates with clinical examination and serum bilirubin values can guide more targeted counselling tools and preventive education programmes. Therefore, it is of interest to evaluate the accuracy of maternal recognition and grading of neonatal jaundice in relation to clinical assessment and timing of presentation.

## Materials and Methods:

This cross-sectional study was done after approval from the Institutional Ethics Committee. Five hundred newborns with clinical neonatal jaundice admitted to the Special Newborn Care Unit (SNCU), Department of Paediatrics, were included after parental consent. Newborns older than 28 days, babies already admitted for other illnesses and those with major congenital malformations were excluded. Antenatal and natal history was taken from the mother or caregiver and recorded in a predesigned proforma. Peripheral venous blood was collected under aseptic precautions at the earliest possible time for serum bilirubin estimation. Blood group and Rh typing and other relevant tests were done in the hospital blood bank and findings were documented. Clinical evaluation of jaundice was done using Kramer's scale [8] in cephalocaudal direction. Visible yellow discolouration of skin due to dermal bilirubin was taken as guide and the extent of icterus was marked according to the defined Kramer zones, which roughly reflect increasing serum bilirubin levels.

## Parental component:

Parents were interviewed for history of jaundice including duration and cephalocaudal extent of yellow discolouration, jaundice in siblings, socioeconomic status, parental occupation and any previous neonatal hospitalisation. At admission, after brief explanation, parents were asked to roughly grade the extent of jaundice according to Kramer's scale for their own baby (Table 1 - see PDF).

## Statistical analysis:

Demographic, history, clinical and laboratory data were entered in a predesigned proforma and analysed. Categorical variables(sex, education, socioeconomic status, parental grading, Kramer zone) were expressed as frequency and percentage and compared using Chi-square test. Continuous variables (age at presentation, serum bilirubin levels) were expressed as mean ± SD and compared using Student's t-test. p < 0.05 was taken as statistically significant. Analysis was done with IBM SPSS Statistics version 16.0.

## Results:

Out of 500 newborns, 330 (66%) were males and 170 (34%) were females. Most babies were full term (70%) and 30% were preterm. Rural background was more common (64%) than urban (36%) and 70% families belonged to middle or upper-lower socioeconomic class. Thus, in this cohort jaundiced newborns were mainly full term, rural and from lower to middle socioeconomic strata (Table 2 - see PDF, Figure 1 - see PDF). More than 80% of newborns (81%) presented within the first 72 hours of life, with 52% reporting between 48-72 hours. Mothers were the first to detect jaundice in 52% cases, fathers in 31% and relatives in 14.6%, while clinicians identified only 2.4% initially. Early reporting (<48 hours) was significantly higher in urban families (p = 0.004) and with maternal literacy (p = 0.022). Previous sibling jaundice (p = 0.031), maternal history of jaundice (p = 0.011) and ABO incompatibility, where 60% reported within 24 hours (p = 0.022), all showed earlier presentation, indicating strong effect of awareness and access (Table 3 - see PDF). Kramer grading showed a significant mismatch between clinician and parental assessment (p = 0.008). Clinicians most often graded babies as Kramer 3 (39.2%) and 4 (24%), while parents also most frequently chose grade 3 (42.6%) but under-reported higher grades. Only 5% of parents selected grade 5 compared to 17% by clinicians, showing that parents tended to underestimate severe jaundice (Table 4 - see PDF). Babies with haemolytic risk factors (ABO or Rh incompatibility) had significantly higher Kramer grades (>3) than those without haemolysis (p = 0.030). Bilirubin encephalopathy occurred more often in neonates of illiterate mothers (p = 0.012), highlighting the impact of maternal education on timely care seeking. Severe jaundice (Kramer >3) was significantly associated with ABO incompatibility (p = 0.022) and infants delivered by LSCS had higher Kramer grades than vaginally delivered babies (p = 0.018) (Table 5 - see PDF, Figure 2 - see PDF). Mean serum bilirubin levels did not differ significantly across Kramer grades (p > 0.05). Bilirubin values were numerically higher in newborns presenting later, but this trend was not statistically significant (p = 0.2145) in the present cohort (Table 6 - see PDF).

## Discussion:

Our study showed male newborns were reporting more with jaundice and this pattern is seen in many regional data also, suggesting some biological and also social influence behind male care-seeking, though the exact reason still not fixed. Similar male dominance was noted in the Ethiopian multi-centre data where boys carried higher risk for significant hyperbilirubinemia [9]. Most of our babies reached the hospital within 72 hours and this early window is important because peak bilirubin commonly rises in the first 3 days. The Somalia multicentre study also highlighted that early presentation was strongly linked with family awareness and prior experience with jaundice at home, matching our findings where mothers with prior jaundice history came faster [10]. In our babies, literacy had a strong effect on how fast families came and how early jaundice was detected. We found educated mothers were the first observers in most cases. A similar pattern was seen in the Ghana 2025 tertiary care study where higher maternal education had a protective effect and delayed encephalopathy was less common in educated groups [11]. Our study also showed parental Kramer assessments were broadly similar to clinician grading in early grades but parents underestimated severe grades like 4 and 5. This aligns with the Indonesian guideline-based evaluation where Kramer score worked well for mild jaundice but was less reliable in darker babies and in higher levels [12]. The Iranian neonatal study also reported that correlation of Kramer with serum bilirubin was moderate and more consistent only in mid-range bilirubin levels, supporting our finding that severe jaundice gets underestimated visually [13]. When we compared our bilirubin trends with global literature, the meta-analysis by Lin et al. showed strong association of early-onset jaundice with hemolytic causes and we also saw ABO-related babies presenting earlier and with higher Kramer grades [14]. Our rural babies presented later than urban families and this delay reflected similar health-system gaps described in the Nigeria referral-centre study where distance and access strongly influenced severity on arrival [15]. Transcutaneous bilirubin and Kramer relation has become more important globally. The Malaysian 2025 comparison trial showed that combining Kramer with TcB reduces need for blood sampling [16]. We also observed that while parental Kramer was fairly consistent in mild jaundice, accuracy dropped as bilirubin levels raised, indirectly agreeing that adding TcB improves reliability. Evidence from the Egyptian 2025 work showed TcB at multiple sites had strong correlation with serum bilirubin even during phototherapy which suggests that visual grading alone is insufficient at higher bilirubin levels [17]. Relation between serum and TcB was also demonstrated strongly in the 2025 South India F1000 study where TcB predicted serum levels well in term and late preterm neonates. This again supports that clinical visual scales alone cannot replace instrument-based tools in higher-risk babies [18]. Our study had a large sample of 500 newborns which allows better observation of patterns in parental recognition. First time in our region we compared parental Kramer with clinician and with biochemical bilirubin, giving a three-way correlation model. We also included very early neonatal age which improves clinical relevance for real-world paediatric practice. It was a single-centre study so generalisation is limited. Clinician visual assessment may have observer differences. We did not include long-term neurodevelopment follow up for babies with high bilirubin. Visual assessment bias could occur due to variation in skin tone and indoor lighting.

## Conclusion:

Neonatal jaundice in our study showed clear pattern where male full-term babies from rural families were reporting more and many were coming late. Educated mothers picked jaundice early and their assessment almost matched clinician judgement, which gives strong hope for community-level training. Parents with past history of jaundice in sibling or mother reacted faster, showing awareness plays big role in preventing severe bilirubin rise. Empowering mothers with simple counselling can easily reduce delayed reporting, encephalopathy risk and avoidable interventions in low-resource settings.

## References

[1] Annisa P (2023). Women Midwives Midwifery..

[2] Tessema M (2024). Front Pediatr..

[3] Choi Y (2025). Children (Basel)..

[4] Pathak A (2022). J Family Med Prim Care..

[5] Alhassan A (2023). J Clin Res Notes..

[6] Fanello C (2023). Am J Trop Med Hyg..

[7] Alinaitwe B (2024). Patient Prefer Adherence..

[8] Dionis I (2021). BMC Pediatr..

[9] Bante AA (2024). Heliyon..

[10] Warsame HA (2024). BMJ Open..

[11] Brobby NAW (2025). BMC Pediatr..

[12] Sampurna MTA (2021). Int J Environ Res Public Health..

[13] Esmaelzadeh-Saeieh S (2022). Iran J Neonatol..

[14] Lin Q (2022). Transl Pediatr..

[15] Ochigbo S (2024). BJOG..

[16] Lim XJ (2025). BMC Pediatr..

[17] Parashar AK (2025). Egypt Pediatr Assoc Gaz..

[18] Selvanathan J (2025). F1000Research..

